# Diagnostic value of MRI for posttreatment surveillance of early-stage (I–II) glottic larynx cancer

**DOI:** 10.1007/s00066-025-02460-6

**Published:** 2025-09-02

**Authors:** Lucas Mose, Emre Korkmaz, Miranda Visini, Roland Giger, Daniel Hendrik Schanne, Olgun Elicin

**Affiliations:** 1 Department of Radiation Oncology, Inselspital, Bern University Hospital and University of Bern, Freiburgstrasse 18, 3010 Bern, Switzerland; 2 Department of Radiation Oncology, Heidelberg University Hospital, Heidelberg, Germany; 3 Department of Urology, Başakşehir Çam and Sakura City Hospital, Istanbul, Turkey; 4 Department of Otorhinolaryngology, Head and Neck Surgery, Inselspital, Bern University Hospital and University of Bern, Bern, Switzerland

**Keywords:** Head and neck neoplasms, Early glottic cancer, Magnetic resonance imaging, Laryngeal neoplasms, Glottic squamous cell carcinoma

## Abstract

**Purpose:**

There is no uniformity across various guidelines in defining the modality and frequency of the follow-up, particularly regarding radiological imaging. The objective is to assess the diagnostic performance of magnetic resonance imaging (MRI)-based posttreatment surveillance for early-stage (I–II) glottic squamous cell carcinoma of the larynx.

**Methods:**

The follow-up of patients diagnosed with glottic squamous cell carcinoma of the larynx, treated with radiotherapy or surgery in curative intent, was analyzed over a period of 2 years posttreatment. MRI diagnostic performance metrics were calculated using clinical and post-MRI endoscopic examinations as the reference standard. MRI sequences included both anatomical and functional imaging, including diffusion-weighted imaging.

**Results:**

In total, 171 eligible MRIs were analyzed in the follow-up. Recurrences were identified with a sensitivity of 75% and a specificity of 99%. However, the positive predictive value of MRI surveillance reflects considerable uncertainty in the diagnosis of recurrences based solely on MRI findings, dropping as low as 21% in sensitivity analyses. Moreover, a notable proportion of MRIs were inconclusive.

**Conclusion:**

MRI demonstrates high specificity and acceptable sensitivity; however, the limited positive predictive value raises concerns regarding its utility as a stand-alone surveillance tool.

**Supplementary Information:**

The online version of this article (10.1007/s00066-025-02460-6) contains supplementary material, which is available to authorized users.

## Introduction

Laryngeal squamous cell carcinoma is one of the most common malignancies of the head and neck with approximately 180,000 newly diagnosed cases and 100,000 deaths annually worldwide [1, 2]. Anatomically, laryngeal carcinomas are classified into supraglottic, glottic, and subglottic tumors, whereby glottic carcinoma represent the largest fraction [3, 4]. Due to the sparse lymphatic drainage of the glottis, lymph node metastases in early glottic laryngeal carcinoma remain rare [5]. Consequently, patients in early stages can be offered successful cure with single-modality approach by means of radiotherapy or surgery. Hereby, both types of treatment were reported to reach comparable local control and long-term survival rates [6–11].

Clinical follow-up typically continues for several years. Shorter time intervals are recommended during the first 2–3 years, in which most recurrences occur [12–15]. However, there is no uniformity across various guidelines in defining the modality and frequency of follow-up, particularly regarding radiological imaging, due to the lack of high-level evidence supporting regular follow-up or patients’ self-referral according signs and symptoms suspicious for recurrence [9, 13, 15–17]. It is also debated whether patients with early-stage glottic carcinoma require radiological follow-up, as recurrences may usually be detected by symptoms (hoarseness) [13, 18]. Hence, this study aims to retrospectively evaluate the performance of the magnetic resonance imaging (MRI)-based tumor surveillance in early-stage (I–II) glottic carcinoma within the first 2 years after the end of treatment.

## Materials and methods

Patients diagnosed with T1 N0 M0 or T2 N0 M0 glottic carcinoma between January 2006 and November 2021 were treated with curative intention at the Inselspital, Bern University Hospital, Bern, Switzerland. Treatment consisted of primary radiotherapy, or transoral surgery with or without adjuvant radiotherapy. Postoperative radiotherapy was administered in case of final R1 or close (< 1 mm) resection margins. All eligible patients were required to be at least 18 years old and have completed treatment according to established guidelines. Follow-up assessments were conducted at the Inselspital, Bern University Hospital, Bern, Switzerland, and included at least one MRI of the neck. Earliest regular scheduled MRI was conducted 3 months after treatment. Our institutional follow-up regimen is summarized in Supplementary Table 1. Patients without a histologically confirmed diagnosis, those who did not complete treatment, individuals whose follow-up occurred at external institutions, or those who did not provide consent for the use of their medical data for research purposes were excluded. All patient, tumor, and procedural characteristics, including medical history and follow-up, were retrospectively collected from the institutional database. The study was approved by the regional ethics committee (Project No. 2023-02215).

**Table 1:** Patient, treatment, and follow-up characteristics

**Patients (*n*** **=** **83)**	
Age (years; median and range)	66.2 (43.4–85.6)
Female	8 (10%)
Male	75 (90%)
**Initial imaging** ^†^ **(*n*** **=** **125)**	
MRI	74 (59%)
CT	39 (31%)
^18^FDG-PET/CT	12 (10%)
**cT stage**^**§**^** (*n*** **=** **83)**	
T1a	57 (69%)
T1b	18 (22%)
T2	8 (10%)
**Involvement of anterior commissure (*n*** **=** **83)**	
Present	42 (51%)
Absent	41 (49%)
**Treatment characteristics (*n*** **=** **83)**	
Radiotherapy	64 (77%)
Surgery	15 (18%)
Surgery and adjuvant radiotherapy	4 (5%)
**Transoral chordectomy**^**#**^** (*n*** **=** **19)**	
Type I	3 (16%)
Type II	10 (53%)
Type III	3 (16%)
Missing data	3 (16%)
**Radiotherapy technique (*n*** **=** **68)**	
VMAT/IMRT	57 (84%)
3D-conformal	11 (16%)
**Radiotherapy fractionations (*n*** **=** **68)**	
34 × 2 Gy	46 (68%)
35 × 2 Gy	10 (15%)
16 × 3.36 Gy	7 (10%)
28 × 2.25 Gy	2 (3%)
33 × 2 Gy	1 (1%)
36 × 2 Gy	1 (1%)
20 × 2.75 Gy	1 (1%)
With elective neck irradiation (25–27 × 2 Gy)	4 (6%)
***Follow-up***	
**MRI (*n*** **=** **199)**	
Scheduled MRI (total)	180 (90%)
Scheduled MRI with preceding endoscopy	7 (4%)
Scheduled MRI without preceding endoscopy	173 (87%)
Nonscheduled MRI upon clinical suspicion	19 (10%)
**Clinical follow-up assessment (*n*** **=** **199)**	
With Endoscopy	171 (86%)

^†^ at the time of initial staging, § according to the AJCC/UICC staging system 8th edition, # chordectomy type according to the European Laryngological Society Classification

*CT* computed tomography, *FDG-PET/CT* ^18^F‑fluorodeoxy-D-glucose positron emission tomography-computed tomography, *Gy* Gray, *IMRT* intensity-modulated radiotherapy, *MRI* magnetic resonance imaging, *n* number, *VMAT* volumetric modulated arc therapy

### Patient, tumor, and procedural characteristics

All in-house MRI acquisitions were performed using intravenous gadolinium-based contrast agents including T1 with and without contrast, T2, turbo inversion recovery magnitude (TIRM), diffusion-weighted imaging and Dixon sequences. A minority of patients were admitted to our department with baseline MRIs from external institutions with varying sequences. However, all these patients subsequently received their follow-up imaging in-house. MRI findings were summarized into “no recurrence”, “local failure (LF)”, “locoregional failure (LRF)”, “second primary malignancy” and “indeterminate result”. The state of being recurrence-free was defined if the endoscopic examination did not reveal recurrence, or if no endoscopy was performed and no clinical signs of recurrence for at least the subsequent 6 months following MRI was documented. To mitigate potential bias in MRI evaluations, prognostic values such as sensitivity, specificity, positive predictive value, negative predictive value and accuracy are calculated based only on regularly scheduled MRIs that have not been preceded by endoscopic evaluations 1 month before or prescribed due to clinical suspicion. Hereby, the calculations for the diagnostic performance were conducted for three scenarios following the Standards for Reporting of Diagnostic Accuracy (STARD) guidelines with MRIs as the index test and clinical examinations as the clinical reference standard [19]: first, scenario in which the cases with indeterminate results are excluded and only the conclusive (positive or negative) MRI results are reported; second, the “worst-case scenario” in which the indeterminate results are counted as false (false positive [FP]/false negative [FN]) results; third, the “best-case scenario” in which the indeterminate results are counted as true (true positive [TP]/true negative [TN]) results. Performance metrics were calculated with the OmniCalculator [20].

## Results

**Fig. 1:**
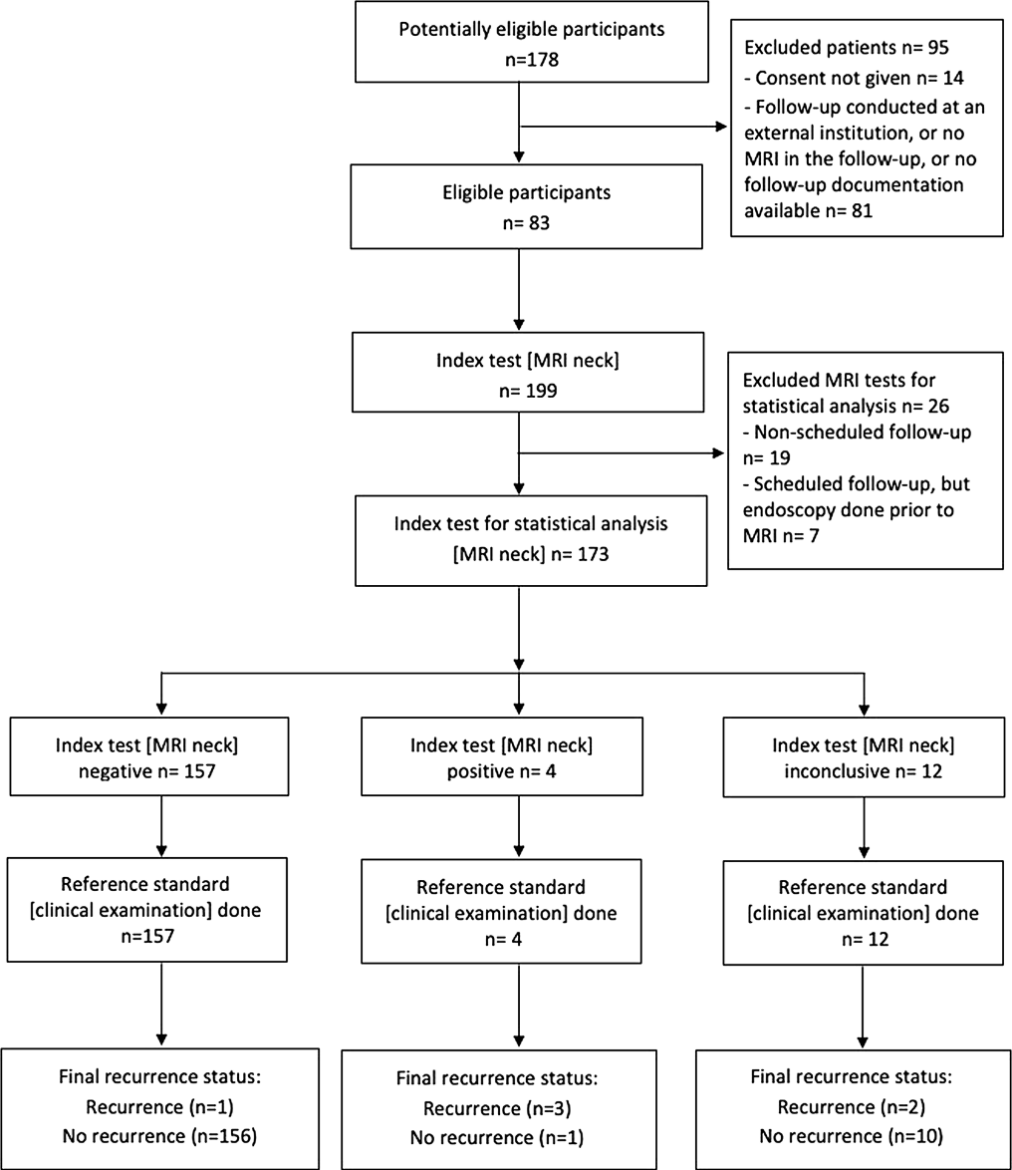
Flowchart for eligible magnetic resonance imaging (MRI) tests for statistical analysis

A total of 178 patients were treated for early-stage glottic cancer; 95 patients did not meet the inclusion criteria (Fig. 1). Of the 83 eligible patients, median age was 66 years with a range from 43 to 86 years. Initial staging was performed with a total of 39 computed tomography (CT) scans and 74 MRI of the neck and 12 ^18^fluorodeoxyglucose-positron emission tomography/computed-tomography (^18^FDG-PET/CT scans). In all, 57, 18 and 18 patients were diagnosed with T1a, T1b, and T2, respectively. Forty-two tumors involved the anterior commissure. Sixty-four patients were treated with radiotherapy, 14 with resection of the primary, 1 with neck dissection and 4 with surgery followed by adjuvant radiotherapy. Radiotherapy was mostly conducted using volumetric arc radiotherapy (VMAT) with total doses ranging between 55 and 72 Gy to the primary via various dose and fractionation regimens. In 4 patients, of which 3 were diagnosed with stage T2 and 1 patient with T1b, the ipsi- and contralateral neck was electively irradiated with a dose of 50–54 Gy. From a total of 199 MRIs, 180 MRIs were conducted according to follow-up schedule within the first 2 years and 19 MRIs were conducted due to clinical suspicion of recurrence. The reasons for unscheduled follow-up examinations were progressive hoarseness (*n* = 7), inspiratory stridor or dyspnea (*n* = 3) as well as cough (*n* = 2), other clinical (*n* = 2) or unknown reasons (*n* = 5). In addition to MRIs, clinical follow-up examinations were performed, 171 with flexible fiberoptic endoscopy and 28 without. Further details on the patient, tumor, and procedural characteristics are provided in Table 1.

Among the 199 clinical follow-up examinations in the 83 patients, 15 failures were diagnosed, all being histologically confirmed. MRI-based results and their classification into TP and TN as well as FP and FN results are reported in Table 2. In the 180 regularly scheduled clinical follow-up examinations, 6 failures were diagnosed. Among the 19 nonscheduled clinical follow-up examinations performed upon clinical suspicion, 1 second metachronous head and neck cancer, 7 local, and 2 locoregional failures were diagnosed. Details on the recurrences, the salvage treatments and oncological follow-up are summarized in Supplementary Table 2, Supplementary Fig. 1, and Supplementary Fig. 2.

**Table 2:** Follow-up results

**All follow-up visits (*n*** **=** **199)**					
	*NR*	*LF*	*LRF*	*2nd Primary*	*Indeterminate result*
MRI-based results	172 (86%)	8 (4%)	4 (2%)	1 (1%)	14 (7%)
Clinical examination-based results	183 (92%)	11 (6%)	4 (2%)	1 (1%)	
	*FN*	*FP*	*TN*	*TP*	*Indeterminate result*
MRI result classification	1 (1%)	2 (1%)	172 (86%)	10 (5%)	14 (7%)
**Scheduled follow-up visits (*n*** **=** **180)**					
	*NR*	*LF*	*LRF*	*2nd Primary*	*Indeterminate result*
MRI-based results	164 (91%)	2 (1%)	2 (1%)	0 (0%)	12 (7%)
Clinical examination-based results	174 (97%)	4 (2%)	2 (1%)	0 (0%)	
	*FN*	*FP*	*TN*	*TP*	*Indeterminate result*
MRI result classification	1 (1%)	1 (1%)	163 (91%)	3 (2%)	12 (7%)
**Scheduled follow-up visits without prior endoscopy (*n*** **=** **173)**					
	*NR*	*LF*	*LRF*	*2nd Primary*	*Indeterminate result*
MRI-based results	157 (91%)	2 (1%)	2 (1%)	0 (0%)	12 (7%)
Clinical examination-based results	167 (97%)	4 (2%)	2 (1%)	0 (0%)	
	*FN*	*FP*	*TN*	*TP*	*Indeterminate result*
MRI result classification	1 (1%)	1 (1%)	156 (90%)	3 (2%)	12 (7%)
**Nonscheduled follow-up visits (*n*** **=** **19)**					
	*NR*	*LF*	*LRF*	*2nd Primary*	*Indeterminate result*
MRI-based results	8 (42%)	6 (32%)	2 (11%)	1 (5%)	2 (11%)
Clinical examination-based results	9 (47%)	7 (37%)	2 (11%)	1 (5%)	
	*FN*	*FP*	*TN*	*TP*	*Indeterminate result*
MRI result classification	0 (0%)	1 (5%)	9 (47%)	7 (37%)	2 (11%)

*FN* false negative, *FP* false positive, *MRI* magnetic resonance imaging, *NR* no recurrence, *LF* local failure, *LRF* locoregional failure, *TN* true negative, *TP* true positive

Of the 180 regular MRIs, 173 were conducted with no preceding flexible fiberoptic endoscopy. The performance metrics were calculated based on these 173 MRIs (Fig. 1) for the three scenarios as described in the “Materials and methods” section. The three recurrences detected as TP by MRI encompassed the following stages: rcT3 rcN0, rcT0 rcN2c and rcT0 rcN3b. Sensitivity and specificity of MRI results were 75% (worst case: 50%, best case: 83%) and 99% (worst case: 93%, best case: 99%), respectively, with an accuracy of 99% (worst case: 92, best case:99%). All values are presented in Table 3. Fourfold tables for the calculations for each scenario are provided in Supplementary Table 3.

**Table 3:** Performance of the regularly scheduled, MRI-based follow-up without prior endoscopy (*n* = 173)

	Scenario including conclusive results only (in %)	Worst-case scenario (in %)	Best-case scenario (in %)
*Sensitivity*	75	50	83
*Specificity*	99	93	99
*Positive predictive value*	75	21	83
*Negative predictive value*	99	98	99
*Accuracy*	99	92	99

*MRI* magnetic resonance imaging

## Discussion

To the best of our knowledge, this is the first study reporting MRI-based surveillance data of a cohort encompassing solely early-stage glottic carcinoma. This distinguishes our study from prior literature on surveillance of head and neck cancer patients, which often report heterogeneous cohorts with various tumor stages and primary cancers [12, 21]. In our findings, MRI surveillance demonstrated acceptable sensitivity (75%) and high specificity (99%) in detecting recurrences, with values ranging between 50 and 83% for sensitivity and between 93 and 99% for specificity depending on how indeterminate results were handled.

Posttherapeutic surveillance in head and neck cancer patients serves primarily to detect recurrences as soon as possible [12, 18]. As the majority of recurrences manifest within the first 2–3 years posttreatment, intensive surveillance is recommended particularly during this period [12–15]. However, the recommendations for imaging modalities and time intervals between follow-ups lack standardized guidelines [9, 13–15, 17]. The effectiveness of posttherapeutic surveillance of head and neck patients is a topic of current debate. There are studies suggesting that routine follow-ups may not provide a survival advantage over self-referral due to suspicious signs and symptoms [12, 17]. For instance, Ritoe et al. analyzed 402 laryngeal cancer patients and found no significant difference in salvage therapy choices, intentions of salvage therapy or survival outcomes among patients whose recurrences were detected during routine follow-ups without symptoms, routine follow-ups with symptoms, or self-referral because of symptoms [22]. The lack of benefit could be attributed to the inevitable poor prognosis associated with recurrences and the limited therapeutic options in case of a recurrence or a second primary malignancy [12, 22, 23]. In contrast, other studies indicate that routine follow-ups may indeed affect survival outcome [24]. Proponents of standardized surveillance in follow-ups argue that asymptomatic recurrences can be detected early in order to avoid further treatment delay [12, 25]. In accordance with this, Hermans et al. found that imaging provides findings of recurrence in laryngeal and hypopharyngeal cancers prior to physical detection [26]. Overall, further research is needed to clarify the most effective approach to posttreatment surveillance in head and neck cancer patients.

Regarding imaging modalities used in surveillance, some experts suggest employing the same imaging method utilized during pretreatment evaluations [12]. For the staging of glottic cancer, Allegra et al. demonstrated superiority of MRI over CT with a stronger correlation between radiological findings and pathological reports [27]. However, data reporting the value of MRI for the stating of laryngeal cancer remain scarce and, thus, inconclusive [28, 29]. Yet, there are advantages of MRI over CT like its avoidance of ionizing radiation exposure for the patient. Additionally, data underscore the advances of MRI over CT in providing enhanced soft tissue contrast and distinguishing between posttreatment changes and cancer recurrence [28, 30, 31]. Nevertheless, it is noteworthy that MRI cannot be conducted in patients with ferromagnetic foreign bodies and dedicated assessment and monitoring has to be done in patients with implanted cardioverter–defibrillators, permanent pacemakers or cochlea implants [28, 29, 32]. MRI results may be affected by respiratory or swallowing motions and FP evaluations can occur due to fibrosis and scar tissue formation [28, 29]. The inclusion of functional MRI sequences such as diffusion-weighted imaging may have contributed to improved diagnostic confidence. However, we did not analyze diagnostic metrics separately for functional versus purely anatomical MRI, which could be explored in future studies with a preplanned protocol.

The aforementioned studies on surveillance often involve heterogeneous head and neck cancers at various stages, potentially leading to distinct recurrence patterns as seen in glottic carcinoma and, therefore, may require different surveillance [17, 33]. In glottic carcinoma, Johansen et al. reported a recurrence pattern, which involved the initial T‑position in 91% [34]. Though the region is accessible for endoscopic examination, recurrences of squamous cell carcinoma predominantly occur beneath intact mucosa where laryngoscopy alone may not detect intricate details such as depth of infiltration, paraglottic invasion, and tumor spread [28, 29, 35]. Hereby, MRI could help evaluating these details [28]. Nonetheless, the diagnosis of recurrences remains paramount, especially in patients with early-stage head and neck cancers, as they may benefit from salvage treatment with superior survival rates compared to those with advanced initial tumor stages [36].

According to our current findings, posttherapeutic surveillance using MRI demonstrates high specificity (up to 99%) and acceptable sensitivity (up to 83%) in the best-case scenario. However, the positive predictive value varied substantially between scenarios, from 21% in the worst-case to 83% in the best-case, raising concerns about the reliability of MRI as a stand-alone test. Approximately one in four patients diagnosed with a recurrence based on MRI findings would in fact be misclassified. This degree of uncertainty necessitates confirmatory clinical and endoscopic examination following every positive MRI result. Given the absence of data on the performance of endoscopic examination in this study, it is difficult to determine how many patients would require both MRI and endoscopic examination to diagnose recurrences, which may have been missed solely by endoscopy. Nonetheless, we expect a considerably high number needed to scan via MRI followed by an endoscopy. This is despite the fact that a notable proportion of MRI results remain inconclusive and some of the recurrences already present with symptoms that would prompt diagnosis regardless of the surveillance modality. It is also worthy to note that only one of the three detected recurrences in our cohort was local, whereas the remaining two were isolated lymph node recurrences.

Due to the limited availability of data on costs and health benefits of MRI examinations in our cohort, and the shortcomings associated with conducting a cost effectiveness analysis based solely on data from a single center/single healthcare setting, a cost-effectiveness analysis was not deemed meaningful [37]. Yet, such an analysis was performed by Zhou et al. on MRI examinations for patients diagnosed with nasopharyngeal carcinoma and treated with definitive radiotherapy [38]. Considering the low recurrence rates in patients with T1/2 nasopharyngeal carcinoma and the costs of MRI examinations, the authors concluded that MRI surveillance for these patients would not be cost-effective [38]. Notably, this setting may be comparable to our cohort, as recurrence rates in patients diagnosed with early-stage laryngeal cancer are low as well. Nevertheless, a dedicated cost-effectiveness analysis would be necessary for early-stage laryngeal cancer.

Our study harbors inherent limitations due to its retrospective nature, most importantly, a selection bias which limits the general applicability of these findings. Another potential bias may have been introduced as physicians conducting endoscopic examinations had access to MRI and their findings, possibly influencing diagnostic assessments. Additionally, the absence of a cohort undergoing solely endoscopic evaluation precludes direct comparison, hindering a comprehensive assessment of the performance of the MRI. Thus, determining the optimal surveillance strategy and the exact number of scans required for effective monitoring remains unclear within the study’s framework.

## Conclusion

The findings of this first magnetic resonance imaging (MRI)-based study data on posttherapeutic surveillance of early glottic carcinoma demonstrate high specificity and acceptable sensitivity. However, due to the rarity of recurrences, interpretation of the positive predictive value of MRI surveillance requires caution. Recurrences may be diagnosed solely based on routine endoscopic examination or due to symptomatic self-referral by the patients. Thus, the added value of MRI is questionable as a sufficiently effective method for surveillance in this context and could potentially be omitted in the follow-up surveillance of early glottic carcinoma. A prospective cohort study is needed to confirm or reject this hypothesis. Until then, we will omit the routine use of MRI surveillance in this patient population at our institution.

## Supplementary Information

Supplementary Fig. 1 Kaplan–Meier curve for local control after salvage treatment

Supplementary Fig. 2 Kaplan–Meier curve for overall survival after salvage treatment

Supplementary Table 1 Recommended follow-up schedule for patients with early-stage laryngeal cancer within the first 2 years after treatment at our institution, Supplementary Table 2 Salvage treatments and oncological outcome of recurrences based on scheduled follow-up, Supplementary Table 3 Cross table for the three scenarios: scenario including conclusive results only, worst-case scenario, best-case scenario

## Abbreviations

CT: computed tomography
FDG-PET/CT: 18F-fluorodeoxy-D-glucose positron emission tomography–computed tomography
FN: false negative
FP: false positive
Gy: Gray
IMRT: intensity-modulated radiotherapy
LF: local failure
LRF: locoregional failure
MRI: magnetic resonance imaging
*n*: number
NR: no recurrence
STARD: Standards for Reporting of Diagnostic Accuracy Studies
TN: true negative
TP: true positive
VMAT: volumetric modulated arc therapy
