# Supplementary material for: Diagnostic value of MRI for posttreatment surveillance of early-stage (I–II) glottic larynx cancer
Source: Strahlenther Onkol. 2025 Sep 2;202(4):372–9. doi: 10.1007/s00066-025-02460-6 (PMC12999828; doi:10.1007/s00066-025-02460-6)
Supplement: Supplementary file 1 — Supplementary Fig. 1 Kaplan–Meier curve for local control after salvage treatment [file 66_2025_2460_MOESM1_ESM.docx]

Supplementary Table 1:
Recommended follow-up schedule for patients with early-stage laryngeal cancer within the first two years after treatment at our institution
Abbreviations: MRI: magnetic resonance imaging, TSH: Thyroid-stimulating hormone, CT: computed tomography

| **Time after treatment in months** | **Follow-Up** |
| --- | --- |
| 3 | - MRI head and neck  - clinical examination |
| 6 | - clinical examination  - in case of radiotherapeutic treatment: TSH-evaluation |
| 9 | - clinical examination |
| 12 | - MRI head and neck  - chest CT scan  - clinical examination - in case of radiotherapeutic treatment: TSH-evaluation |
| 15 | - clinical examination |
| 18 | - clinical examination |
| 21 | - clinical examination |
| 24 | - MRI head and neck  - chest CT scan  - clinical examination - in case of radiotherapeutic treatment: TSH-evaluation |

Supplementary table 2: Salvage treatments and oncological outcome of recurrences based on scheduled follow-up

| **TNM at**  **initial diagnosis** | **First treatment** | **TNM stage at recurrence** | **Second treatment** | **LC time in months after salvage treatment** | **LC after salvage treatment**  **1= no recurrence**  **2= recurrence** | **Survival after salvage treatment -**  **FU in months** | **Last known status**  **1= alive**  **2= dead** |
| --- | --- | --- | --- | --- | --- | --- | --- |
| **Recurrences diagnosed with scheduled follow-up** | | | | | | | |
| cT1a cN0 cM0 | Chordectomy type III, | rcT2 rcN0 cM0 | Radiotherapy (70 Gy) | 50 | 1 | 53 | 2 |
| cT1a cN0 cM0 | Radiotherapy (16 x 3,63 Gy, 58 Gy) | rcT0 rcN2c rcM0 | Induction chemotherapy,  Neck dissection,  Postoperative radiotherapy (66 Gy) with concomitant cetuximab | 53 | 1 | 53 | 1 |
| cT1b cN0 cM0 | Radiotherapy (34 x 2Gy, 68 Gy) | rcT3 rcN0 rcM0 | Palliative immuno-chemotherapy (cetuximab/ 5-fluoracil/ calciumfolinat) | NA | NA | 12 | 2 |
| cT2 cN0 cM0 | Radiotherapy (35 x 2 Gy,  70 Gy) | rcT2 rcN0 cM0 | Chordectomy type IV | 13 | 2 | 18 | 2 |
| cT1a cN0 cM0 | Radiotherapy (34 x 2 Gy, 68 Gy) | rcT3 rcN0 rcM0 | Re-irradiation  (33 x 2 Gy, 66 Gy) | 5 | 1 | 5 | 1 |
| cT1a cN0 cM0 | Radiotherapy (34 x 2 Gy, 68 Gy) | rcT0 rcN3b rcM0 | Neck dissection | 51 | 2 | 65 | 1 |
| **Recurrences diagnosed with self-referral** | | | | | | | |
| cT1a cN0 cM0 | Chordectomy type II | rcT0 rcN1 rcM0 | Neck dissection | 48 | 1 | 48 | 1 |
| cT1a cN0 cM0 | Chordectomy type II | rcT1 rcN0 rcM0,  or  second carcinoma | Chordectomy type II | 82 | 1 | 2 | 2 |
| cT1a cN0 cM0 | Radiotherapy (34 x 2 Gy, 68 Gy) | rpT2 rcN0 rcM0 | Hemilaryngectomy | 61 | 1 | 61 | 1 |
| cT1a cN0 cM0 | Radiotherapy (35 x 2 Gy, 70 Gy) | rcT1 rcN0 rcM0 | Chordectomy type IV | 26 | 2 | 90 | 1 |
| cT1a cN0 cM0 | Radiotherapy (34 x 2 Gy, 68 Gy) | rcT1 rcN0 rcM0 | Chordectomy type IV with  neck-dissection | 4 | 2 | 80 | 1 |
| cT1b cN0 cM0 | Radiotherapy (34 x 2 Gy, 68 Gy) | rp4a rcN0 rcM0 | Laryngectomy with neck-dissection and adjuvant radiotherapy (66 Gy) | 120 | 1 | 135 | 2 |
| cT1b cN0 cM0 | Radiotherapy (35 x 2 Gy,  70 Gy) | rcT2 rcN0 cM0 | Hemilaryngectomy with adjuvant immuno-chemotherapy (pembrolizumab, carboplatin) | NA | NA | 43 | 1 |
| cT1 cN0 cM0 | Radiotherapy (68 Gy) | rcT4 cN1 cM0 | Laryngectomy with neck dissection R1/2,  adjuvant radiotherapy (66 Gy) with concomitant cetuximab | 9 | 2 | 12 | 1 |
| cT1a cN0 cM0 | Radiotherapy (34 x 2 Gy, 68 Gy) | rcT3 rcN0 rcM0 | Laryngectomy with neck dissection | 90 | 1 | 92 | 2 |

Supplementary table 3: Cross table for the three scenarios: Scenario including conclusive results only, worst case scenario, best case scenario

| ***Scenario including conclusive results only*** | | |
| --- | --- | --- |
| ***N total = 161*** | ***Histological proven recurrence (n= 4)*** | ***No recurrence  (n= 157)*** |
| ***MRI indicates recurrence (n= 4)*** | *True positive:*  *3* | *False positive:*  *1* |
| ***MRI indicates no recurrence***  ***(n= 157)*** | *False negative:*  *1* | *True negative:*  *156* |
| ***Worst case scenario*** | | |
| ***N total = 173*** | ***Histological proven recurrence (n= 6)*** | ***No recurrence  (n= 167)*** |
| ***MRI indicates recurrence (n= 14)*** | *True positive:*  *3* | *False positive:*  *11*  *(1 + 10)* |
| ***MRI indicates no recurrence***  ***(n= 159)*** | *False negative:*  *3*  *(1 + 2)* | *True negative:*  *156* |
| ***Best case scenario*** | | |
| ***N total = 173*** | ***Histological proven recurrence (n=6)*** | ***No recurrence  (n= 167)*** |
| ***MRI indicates recurrence (n= 6)*** | *True positive:*  *5*  *(3 + 2)* | *False positive:*  *1* |
| ***MRI indicates***  ***no recurrence***  ***(n= 167)*** | *False negative:*  *1* | *True negative: 166*  *(156 + 10)* |
