# Supplementary figures and images for: Diagnostic value of MRI for posttreatment surveillance of early-stage (I–II) glottic larynx cancer
Source: Strahlenther Onkol. 2025 Sep 2;202(4):372–9. doi: 10.1007/s00066-025-02460-6 (PMC12999828; doi:10.1007/s00066-025-02460-6)

## Local control after salvage treatment

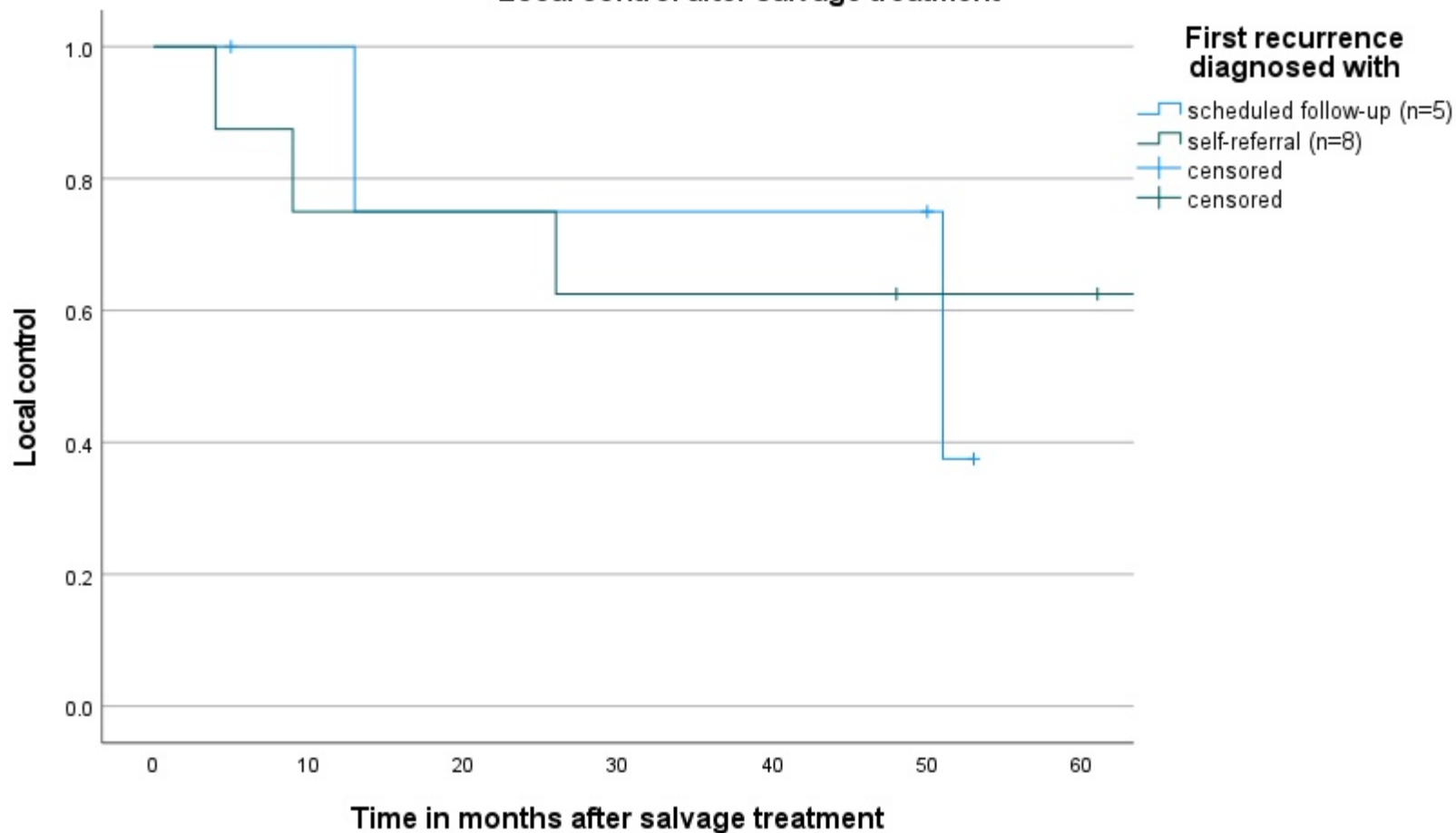

Supplement: Supplementary file 2 — Supplementary Fig. 2 Kaplan–Meier curve for overall survival after salvage treatment [file 66_2025_2460_MOESM2_ESM.pdf]

## Overall survival after salvage treatment

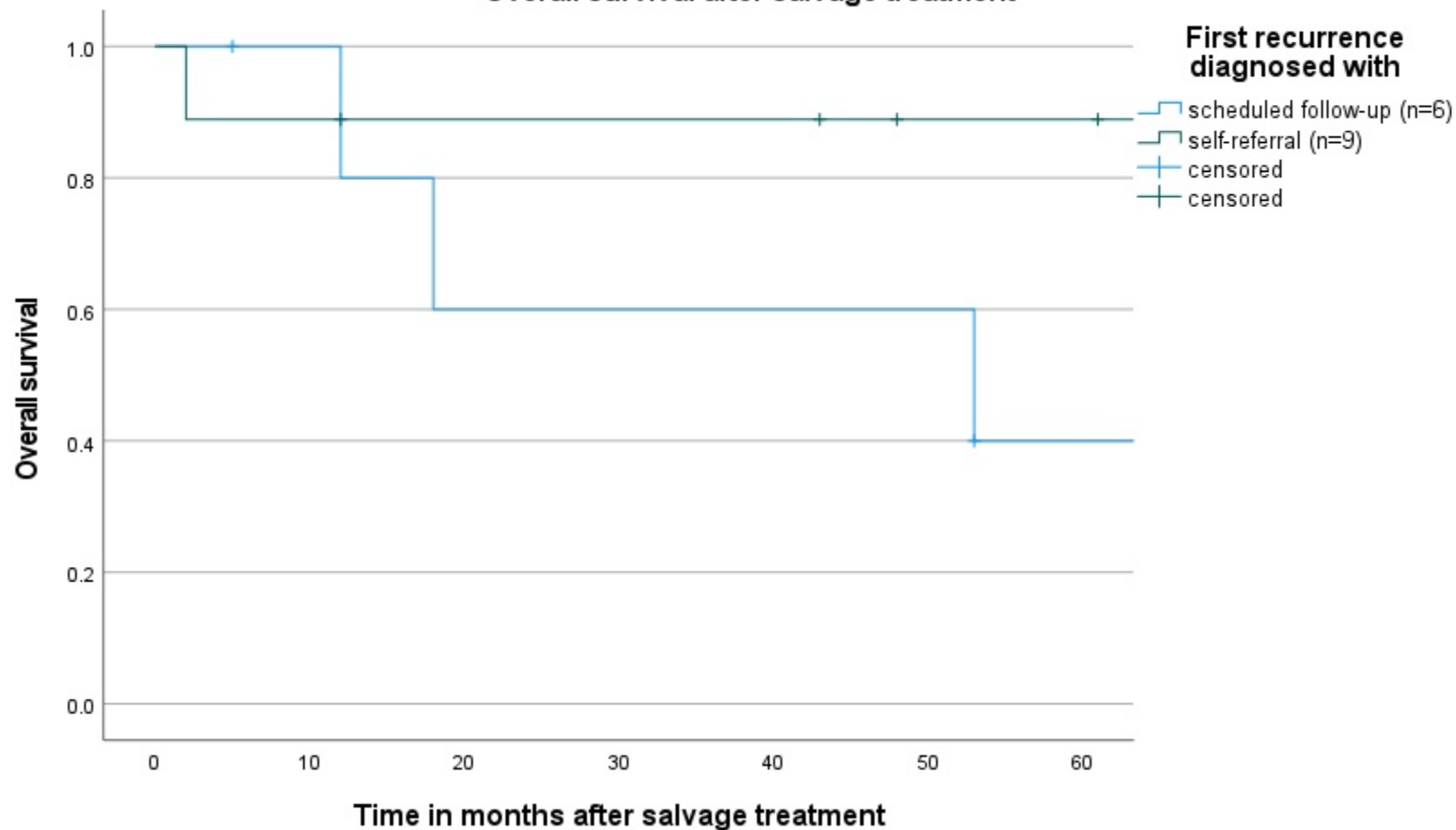

Supplement: Supplementary file 3 — Supplementary Table 1 Recommended follow-up schedule for patients with early-stage laryngeal cancer within the first 2 years after treatment at our institution, Supplementary Table 2 Salvage treatments and oncological outcome of recurrences based on scheduled follow-up, Supplementary Table 3 Cross table for the three scenarios: scenario including conclusive results only, worst-case scenario, best-case scenario [file 66_2025_2460_MOESM3_ESM.pdf]
